# Rethinking thecodonty: the influence of two centuries of comparative dental anatomy on our understanding of tooth evolution

**DOI:** 10.1098/rsbl.2025.0316

**Published:** 2025-09-24

**Authors:** Gabriel Mestriner, Gregory F. Funston, Júlio C. A. Marsola, Sterling J. Nesbitt, Max C. Langer, David C. Evans, Aaron R. H. LeBlanc

**Affiliations:** ^1^ Biologia, Universidade de São Paulo, Ribeirão Preto, São Paulo, Brazil; ^2^ Ecology and Evolutionary Biology, University of Toronto, Toronto, Ontario, Canada; ^3^ Royal Ontario Museum Department of Natural History, Toronto, Ontario, Canada; ^4^ Anatomical Sciences, Stony Brook University, Stony Brook, NY, USA; ^5^ Earth and Planetary Sciences, University of California Davis, Davis, CA, USA; ^6^ Universidade Tecnológica Federal do Paraná - Campus Dois Vizinhos, Dois Vizinhos, Paraná, Brazil; ^7^ Geosciences, Virginia Polytechnic Institute and State University, Blacksburg, VA, USA; ^8^ Faculty of Dentistry, Oral and Craniofacial Sciences, King's College London, London, UK

**Keywords:** thecodont, ankylothecodont, gomphosis, ankylosis, tooth attachment, tooth implantation, bone of attachment, alveolar bone, periodontal ligament, cellular cementum

## Abstract

‘Thecodont’ refers to teeth implanted in sockets within the jaw, a condition traditionally associated with living mammals and crocodylians, which also coincidentally have teeth attached by ligaments to the socket walls (gomphosis). For over a century, the bony periodontium of many other amniotes has been described as a single tissue, ‘bone of attachment’, causing confusion over dental tissue homology. The conventional definitions of ‘thecodonty’ exclude species with fused teeth (‘ankylothecodonts’), implying a fundamental difference between mammals, crocodylians and most other vertebrates. However, the stereotypically ‘thecodont’ attachment tissues have been discovered in representatives of all major amniote clades, showing that gomphosis and ankylosis likely stem from heterochronic changes in the timing and extent of cementum and alveolar bone mineralization. This challenges (i) previous hypotheses regarding the evolution of the amniote periodontium, (ii) the ‘bone of attachment’ paradigm, and (iii) the significance of ‘thecodonty’. We suggest a new nomenclatural approach that incorporates recent histological and evolutionary research and divides thecodonty into anatomical categories to clarify their origin and evolution. We propose the terms anisothecodont and isothecodont to denote, respectively, asymmetric and symmetric implantation of teeth in their sockets. Regardless of the geometry of the connection, we propose using ankylosis and gomphosis to denote the mode of tooth attachment.

## 1. Historical context and tooth attachment nomenclature

The study of teeth provides pivotal data for interpreting the diet and ecology of extinct vertebrates (e.g. [1–3]). In particular, the interaction between teeth and their respective jaw bones can be described by two characteristics: (i) tooth implantation, which categorizes teeth by their spatial and geometrical relations to the bone and (ii) tooth attachment, which distinguishes how teeth are held in their sockets, as either fused to the bone (ankylosis) or suspended by a ligament (gomphosis). Histological investigations have yielded valuable insights into the evolution of tooth attachment and implantation in several amniote clades [4–10]. For example, recent studies shed light on the evolutionary history of the mammalian tooth attachment system, showing that its origin can be traced back to stem-mammal clades (e.g. non-mammalian synapsids) from the Permian and Triassic periods.

**Figure 1.**
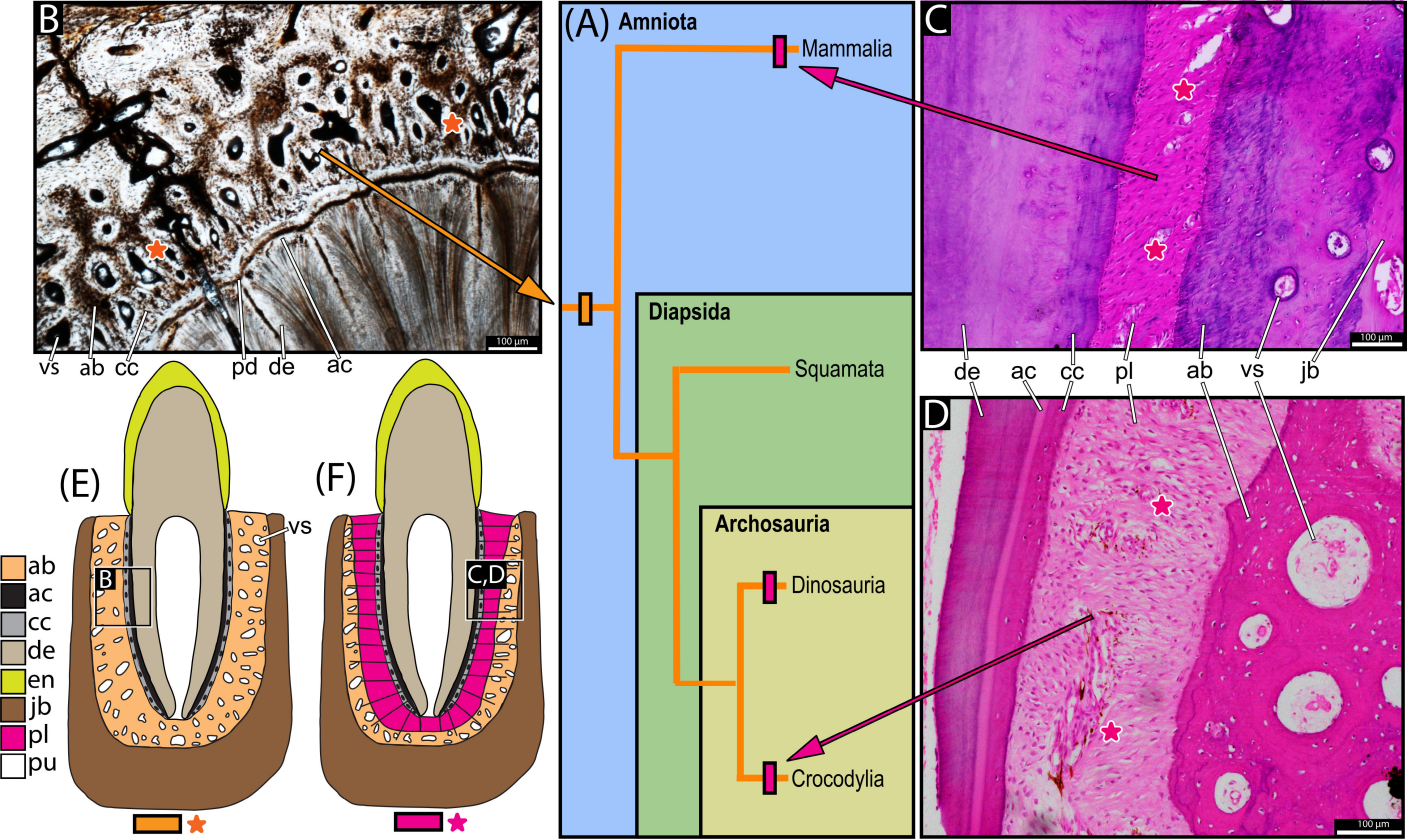
(A) Simplified phylogeny of Amniota depicting ankylosis as the ancestral tooth attachment condition (orange bar) and the independent origin of gomphosis in distantly related clades (pink bar). (B) Tooth attachment tissues of a diadectid (TMM 43628-3: stem or early amniote), showing ankylosis. (C) Tooth attachment tissues of a human tooth root (from the *Berkovitz dental histology teaching collection, King’s College London*) in its socket, showing gomphosis. Mirrored left-to-right to match panel (D). (D) Tooth attachment tissues of *Crocodylus* sp. (Crocodylia; from *Dental Histology Teaching Collection, King’s College London*) showing gomphosis. (E) Schematic of the periodontium of an ankylosed tooth. (F) Schematic of the periodontium of a tooth with gomphosis. Notes: panels (E,F) modified from [12,17]. Panels (C,D): histological section stained with haematoxylin and eosin. ab, Alveolar bone; ac, acellular cementum; cc, cellular cementum; de, dentine; en, enamel; jb, jaw bone; pd, plicidentine; pl, periodontal ligament; pu, pulp cavity; vs, vessel space. Orange colour bar/star indicates ankylosis below panel (E); pink colour bar/star indicates gomphosis below panel (F). Observation: here, we portray diadectids as an example of early amniote/stem-amniote, based on the more widely supported phylogenetic hypotheses [18–20], while acknowledging that alternative placements have been proposed [21]. In any case, multiple studies suggest ankylosis as the ancestral condition for tooth attachment among amniotes [17,22–24], so that any early amniote/stem-amniote could serve as an example here.

Within the category of tooth implantation, four classical types describe the spatial relationship between teeth and the jaw bone [11–13]: acrodonty, pleurodonty, aulacodonty and thecodonty. ‘Acrodont’ teeth are attached to the crest or margin of the jaw, as seen in some lepidosaurs (i.e. rhynchocephalians and some squamates). ‘Pleurodont’ teeth are attached to the lingual side of the lateral jaw wall (the pleura), a condition common in most lepidosaurs. ‘Aulacodonty’ is characterized by teeth set in a continuous groove rather than in discrete sockets, where the groove’s depth is at least equal to the height of the crown. ‘Aulacodonty’ was originally defined in ichthyosaurs [14], but it is also found in hatchling and juvenile crocodylians [15,16]. Finally, ‘thecodonty’ traditionally refers to a socketed tooth implantation mode in which the four-walled sockets are deep and symmetric, associated most often with the condition seen in mammals and crocodylians (figure 1). Coincidentally, both groups also bear teeth suspended in their sockets by soft ligaments. For this reason, conventional definitions of ‘thecodonty’ exclude forms with deep sockets if their teeth are ankylosed to the bone, the bearers of which are known as ‘ankylothecodonts’. However, when Richard Owen coined ‘thecodont’ in his book *Odontography*, it was not intended to designate a kind of tooth implantation, but rather a group of extinct reptiles (‘Thecodont Lacertilians’) that, along with other forms such as crocodylians and mammals, possessed teeth ‘implanted in sockets, either loosely or confluent with the bony walls’ [25, p. 266]. Owen [25] grouped dinosaurs, pterosaurs, plesiosaurs, ichthyosaurs, as well as one of the oldest-known archosauromorphs, *Protorosaurus speneri*, into his ‘Thecodontia’, forming what is now recognized as a polyphyletic array of distantly related reptiles. By the twentieth century (e.g. [26,27]), ‘Thecodontia’ had been re-defined as a grade of reptiles composed of archosaurs and close relatives (e.g. stem-archosaurs) other than crocodylians, pterosaurs and dinosaurs, and the term was ultimately abandoned during the cladistic revolution in palaeontology [28,29].

**Figure 2.**
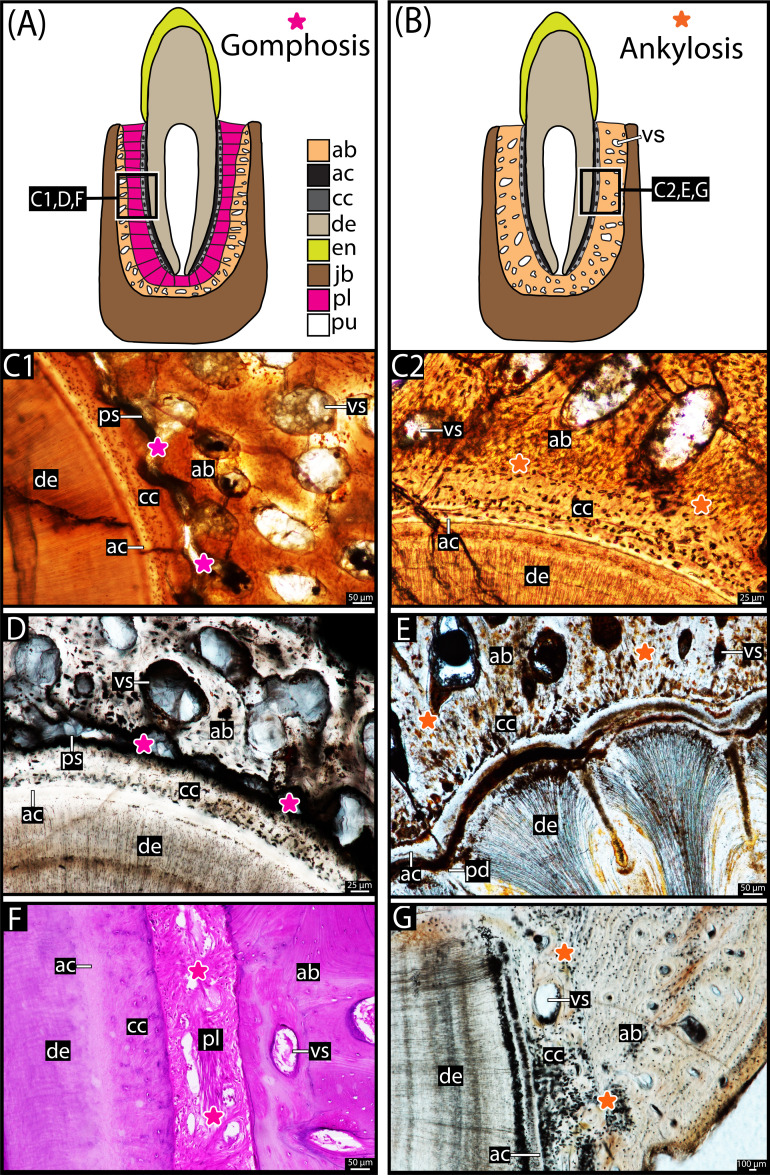
Comparison of dental tissues: gomphosis (A,C1,D,F) versus ankylosis (B,C2,E,G). (A) Schematic of the periodontium of a tooth with gomphosis. (B) Schematic of the periodontium of an ankylosed tooth. (C) Tooth attachment tissues of a specimen of *Eucoelophysis baldwini* (GR 1072, see [9]: Silesauridae, Dinosauriformes), showing gomphosis (C1) in one tooth (note the presence of the periodontal space) and ankylosis (C2) in another (periodontal space completely mineralized), both in the same jaw. (D) Tooth attachment tissues of *Coelophysis bauri* (CM 87671: Theropoda), showing gomphosis (presence of the periodontal space). (E) Tooth attachment tissues of a diadectid (TMM 43628-3: stem or early amniote), showing ankylosis (periodontal space completely mineralized). (F) Tooth attachment tissues of a human tooth root in its socket (from *Berkovitz dental histology teaching collection, King’s College London*), showing gomphosis (presence of an extensive periodontal ligament filling the periodontal space). (G) Tooth attachment tissues of *Captorhinus* (ROM 66861: early amniote), showing ankylosis (periodontal space completely mineralized). Note for panel (F): histological section stained with haematoxylin and eosin. ab, Alveolar bone; ac, acellular cementum; cc, cellular cementum; de, dentine; en, enamel; jb, jaw bone; pd, plicidentine; pl, periodontal ligament; pu, pulp cavity; vs, vessel space. Pink stars indicate the presence of gomphosis; orange stars indicate the presence of ankylosis, highlighting points where the alveolar bone and cellular cementum connect, completely entombing the periodontal ligament/space.

In his *A manual of dental anatomy*, the English dentist Charles Tomes [30] re-interpreted Owen’s observations and proposed a nomenclatural split for teeth suspended by ligaments and those fused to the jaw. He named ‘bone of attachment’ [30, p. 208] as the tissue responsible for ankylosing teeth in many reptiles, which was of unknown homology to the three tooth attachment tissues—cellular cementum, periodontal ligament and alveolar bone—found in mammals, including humans [30, p. 213]. However, recent studies have revealed that these three attachment tissues were ancestrally present in all major amniote clades, even in species with ankylosed teeth (figure 2 and electronic supplementary material, figure 1). By studying the periodontium of snakes [31], mosasaurs [5,32], ichthyosaurs [33] and extant lizards [34], researchers first recognized cementum and alveolar bone, not ‘bone of attachment’, in ankylosed teeth. More recently, following this new perspective, it was found that the differences between tooth attachment modes arise not from de novo evolution of tooth attachment tissues, but from heterochronic changes in the timing and extent of mineralization of the alveolar bone and cementum, with ankylosis representing the last phase of dental ontogeny and the ancestral condition of Amniota [8,9]. Under this model, the stereotypically mammalian tooth attachment tissues are found in all amniotes, but are variably mineralized when a tooth is fully functional; mammals and crocodylians are simply paedomorphic in their tooth attachment mode relative to their ancestors, retaining teeth at an earlier stage and preventing them from fusing to the jaw.

Interestingly, Edmund [35] correctly assumed that in taxa then referred to as ‘true thecodonts’ (i.e. mammals and crocodylians), the permanent presence of soft tissue between the cementum and the alveolus resulted from a diminution in the deposition of calcified tissue between them. In contrast, he noted that ancestrally, in the ‘protothecodonts’ (a term he used synonymously with ‘ankylothecodont’), the deposition of calcified tissue was ‘carried to completion, resulting in ankylosis’ [35, p. 129]. Edmund’s assumptions were later confirmed by recent studies [5,8,9,32,33], which support the amniote ancestral presence of ankylosis (figure 1B) as a result of continuous mineralization of the soft tissues by the surrounding alveolar bone and/or cellular cementum. This contrasts with the condition in taxa with gomphosis—such as mammals and crocodylians (figure 1C,D)—in which the mineralization process is heterochronically delayed or suppressed, thereby retaining a permanent soft tissue attachment between the cementum and alveolus, just as Edmund had proposed [8,9].

## 2. Tooth attachment tissues

Under this new model, the diverse forms of tooth attachment in amniotes simply include different arrangements of the same symplesiomorphic periodontal tissues: cementum, periodontal ligament and alveolar bone (figure 2) [5,7–9,13,17,32–34,36–40]. Thanks in large part to work on mammalian tooth development, we know that the periodontal tissues are derived from the dental follicle, an aggregate of ectomesenchymal cells that surrounds the developing tooth bud [7,17,41–43]. The cementum (figure 2 and electronic supplementary material, figure 1) is the calcified layer of connective tissue that covers the tooth root, divided into acellular and cellular layers [5]. The acellular layer is directly attached to the root dentine and the cellular layer contains many cementocytes that sustain the cementum matrix and anchor fibres of the periodontal ligament [5] in many species (in humans, for example, the acellular cementum can also fulfil this function; see [13]). The periodontal ligament (figures 1C,D, 2F and electronic supplementary material, figure 1D) is an unmineralized network of collagen fibre bundles, fibroblasts, sensory receptors and other cellular components that suspend the tooth within its socket [44]. These ligament fibres perforate the alveolar bone layer and the cellular cementum coating the tooth root. The dentine portion of the tooth—coated by cementum—corresponds to the root of mammal and archosaur teeth [5]. Very common in early amniotes (figures 1B, 2E) but also found in squamates and ichthyosaurs, dentine infolds to form a tissue called plicidentine (see [37] for a review). In addition to providing adaptive advantages—such as increased strength and flexibility at the tooth base, and a greater surface area for tooth attachment tissues [11,37]—this structure has also recently been linked to the origin of snake venom fangs, where a large plicidentine fold develops early in tooth ontogeny and has been repurposed to form a venom groove in some groups [45].

The periodontal ligament supports teeth against occlusal forces, serving a cushioning function through different arrangements of ligament fibre bundles around the tooth root and socket, and a mechanosensory role through innervation from small branches of the trigeminal nerve [46]. In this way, it provides a flexible attachment for the tooth into the alveolar bone, facilitating post-eruptive tooth movements and assisting as a sensory system [47]. Sharpey’s fibres constitute the segment of the periodontal ligament embedded in both the cementum and the alveolar bone, which can be identified in fossils (electronic supplementary material, figure 1B2). These fibres are composed of completely or partially mineralized collagen fibres [17]. In fossils, the ligament itself is not preserved, but the periodontal space that once housed its collagen fibres is identifiable between the tooth root and the surrounding alveolar bone (figure 2C1,D) [7–9,39]. The final component of the periodontium is the alveolar bone (figure 2 and electronic supplementary material, figure 1), which shapes the tooth socket and is formed alongside each new tooth, with each tooth forming its own socket into which it attaches [44].

The wall of bone that separates each tooth socket is called ‘interdental bone’ [5,7,13,31]. The presence of interdental partitions can be ontogenetically variable (e.g. crocodylians and squamates [13,15,43]), and their histological composition can change through ontogeny [13]. For instance, in young crocodylians, these structures are initially formed by jawbone along the anterior region of the dentition, and alveolar bone in posterior region of the jaw [13,15]. In species that do not replace their teeth, like rhynchosaurs, the jawbone serves as interdental bone throughout life [48]. In species that replace their teeth, successive erosion and deposition of alveolar bone results in mineralized partitions consisting mainly of older fragments of alveolar bone from previous generations of teeth [17], and may also include remnants of dentine that are not fully resorbed during successive tooth replacement events [7,9]. In some archosaurs (e.g. dinosaurs), the bone in this region forms a lingual extension between adjacent teeth, known as the ‘interdental plate’ [7]. It is worth noting that neither the interdental plate nor the interdental bone constitutes a distinct tooth attachment tissue or defines the tooth socket itself, as they are not sites of periodontal ligament attachment [7], but accumulations that result from the combination of tooth replacement events and tooth drift [31]. For archosaurs and close relatives, the term ‘interdental unit’ has been proposed as an alternative name, because the interdental plate can be understood as a lingual extension of the interdental bone and has been mistakenly described as a distinct, non-homologous structure [49].

The geometry of the connection of the tooth to the jaw (implantation) is influenced by the shape and persistence of Hertwig's epithelial root sheath (HERS) cells, a bilayered epithelial structure derived from the cervical loop of the enamel organ—the apical portion where the inner and outer enamel epithelium meet. Whereas the enamel organ forms the crown and gives rise to ameloblasts (which produce enamel), the cervical loop elongates apically and gives rise to HERS, guiding root morphogenesis. HERS plays a crucial role in root development by determining its shape and inducing the differentiation of ectomesenchymal cells from the dental papilla into odontoblasts, which subsequently deposit root dentine [36,50,51]. Also, HERS can remain partially (unilaterally) intact surrounding the root, as in squamates (lizards and snakes), or completely disassociated into clusters of epithelial cells, named epithelial rests of Malassez, as in crocodylians and mammals [36]. When HERS disassociates, the cementum and the periodontal ligament start to form along the root surface, moulding the geometry of the tooth root connection to the bone (see topic 6.7 and fig. 6.9 of [13]). When HERS remains intact, as it does in squamates, it acts as a physical barrier to periodontal tissue formation [13], leading to the development of more asymmetrical forms of implantation, as seen in some pleurodont lizards [43,52]. On the other hand, when HERS is broken around the entire tooth root, the periodontal tissues connect to the whole surface of the tooth below the crown, as in crocodylians, synapsids and dinosaurs. Therefore, the transience and position of HERS influence tooth implantation modes, whereas the degree of mineralization of the periodontal tissues through its development dictates tooth attachment modes.

## 3. Deconstructing thecodonty

**Figure 3.**
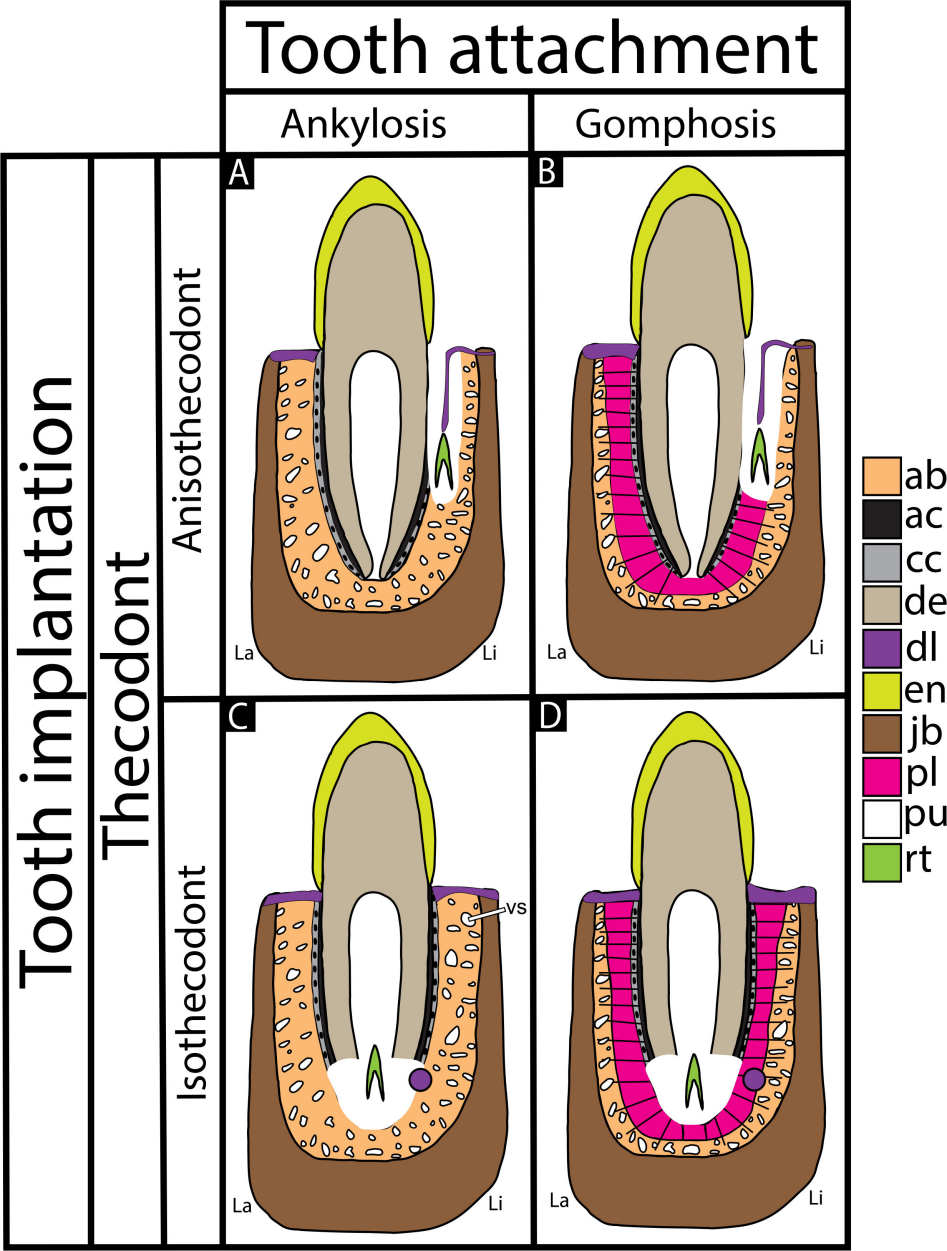
Tooth attachment (ankylosis and gomphosis) and tooth implantation (anisothecodont and isothecodont) categories. (A) Schematic of a thecodont tooth with ankylosis as the mode of tooth attachment, and anisothecodont tooth implantation. (B) Schematic of a thecodont tooth with gomphosis as the mode of tooth attachment, and anisothecodont tooth implantation. (C) Schematic of a thecodont tooth with ankylosis as the mode of tooth attachment, and isothecodont tooth implantation. (D) Schematic of a thecodont tooth with gomphosis as the mode of tooth attachment, and isothecodont tooth implantation. ab, Alveolar bone; ac, acellular cementum; cc, cellular cementum; de, dentine; dl, dental lamina; en, enamel; jb, jaw bone; la, labial; li, lingual; pl, periodontal ligament; pu, pulp cavity; rt, replacement tooth; vs, vessel space.

‘Thecodont’ has always been a taxonomically loaded term, but it has also been used to describe a wide range of taxa over the last 150 years. The long-established (but not the original) tradition that considers crocodylians and mammals to be the true ‘thecodont’ animals implies that both independently acquired a ‘more complex’ form of tooth attachment with three types of attachment tissues. This has been recently challenged by the identification of those three periodontal tissues forming the so-called ‘bone of attachment’ of most reptiles (figure 2 and electronic supplementary material, figure 1) [7–9,12,39,43,45,53]. Moreover, with the ankylosis versus gomphosis dichotomy better understood as stemming from heterochronic changes [8,9], the identical forms of tooth attachment in crocodylians, dinosaurs, and mammals (figure 1A) result not from repeated convergent evolution of these tissues, but from heterochronic delays in the ankylosis. This means that the traditional ‘thecodonts’ are simply retaining teeth attached by ligaments throughout the development of each tooth [8,9]. Hence, gomphosis represents a paedomorphic attribute relative to ankylosis, with every single tooth undergoing gomphosis during its early development. Ankylosis, on the other hand, only represents the last phase of dental ontogeny (figs 3–4 of [8], fig. 16 of [9]), in which ligaments are calcified by alveolar bone and/or cellular cementum growth around the tooth root [5,8,9,35].

When Richard Owen first defined ‘thecodont’, he included both teeth that are fused and those held in place by ligaments. This raises an issue with the historical baggage of the term, used more recently to denote both implantation and attachment modes (e.g. [4]), which are not dependent on one another. Yet, restricting ‘thecodonty’ only to animals with unfused teeth oversimplifies a more complex evolutionary scenario, because it is evident that the presence or absence of ankylosis solely results from shifts in dental ontogeny. The delay in the periodontal ligament mineralization is a unique paedomorphic condition [8,9], but there is no structural distinction (in terms of the identity of the tissues) between a dentition characterized by rapid mineralization (leading to a shorter ligamentous phase and earlier ankylosis) and a dentition that perpetually maintains the ligaments intact (figure 2 and electronic supplementary material, figure 1). What we recommend here is a return to Owen’s original definition.

## 4. Reassessing tooth attachment and implantation terminology

Both thecodont and ‘ankylothecodont’ teeth are equally embedded within distinct sockets, and the tissues forming their periodontium are the same (figure 2 and electronic supplementary material, figure 1). Accordingly, we propose a nomenclatural approach in which ‘thecodonty’ *sensu* Owen ([25], see also [5,7,12]), refers only to the implantation and can be applied to both modes of tooth attachment. Hence, researchers should use ‘thecodont’ only in reference to the presence of teeth that are implanted within sockets in the jaws, without connotation to tooth attachment patterns or even the symmetry or depth of the implantation (figure 3). In fact, differences between symmetrical and asymmetrical alveoli are likely associated with the position of the dental lamina (odontogenetic organ [7,17,39,54–56]), which will form a new tooth during a replacement event and, possibly, HERS. The dental lamina is always positioned lingually along the jaws in amniotes, but when it is attached to the overlying gum line (figure 3A,B), the resulting implantation will be asymmetrical; this is likely ancestral to Amniota [39,54,56]. This asymmetry also creates a taller wall of alveolar bone (or jawbone) along the labial side as the new tooth develops lingually, resorbing mostly the lingual side of the jaw (figure 3A,B), as seen in dinosaurs and most archosaurs [7,9]. In contrast, in groups with the dental lamina buried deep within the jaw (e.g. crocodylians; electronic supplementary material, figure 1C) and disconnected from the overlying gum tissue (figure 3C,D) [7,39,55], the new tooth will spend more time underneath the functional tooth, and surrounding bony walls will be more symmetrical because of more even tooth resorption and, more importantly, because the dental lamina no longer forms a solid wall of epithelium along the lingual side, thereby allowing the lingual side of the tooth to also attach to the jaws (figure 3C,D).

We suggest distinguishing tooth implantation and attachment as separate anatomical categories, with implantation regarded as the geometry of the connection between the tooth and the socket, and attachment regarded as the degree of mineralization of the periodontal ligament. For implantation, we propose the adoption of the new terms anisothecodont (figure 3A,B) and isothecodont (figure 3C,D) to denote, respectively, asymmetric and symmetric alveoli. For attachment, we propose the use of ankylosis (figure 3A,C) and gomphosis (figure 3B,D) to distinguish, respectively, animals with fused teeth from those with an intact periodontal ligament.

In contrast with the idea that thecodonty should be applied only to teeth set in symmetrical alveoli at least as deep as the height of the crowns (i.e. ‘genuine alveoli’ [4,12,26,57,58]), we also regard teeth set in asymmetrical alveoli as thecodonts. Indeed, Bertin *et al*. [12] would not consider hatchling crocodylians thecodonts because the position of their dental lamina (attached to the overlying gum tissue, as in figure 3B) results in a jaw morphology with higher labial walls [7,17,54–56]. It is only in later stages that young crocodylians begin to progressively develop a more symmetrical tooth implantation (dental lamina unattached to the overlying gum tissue, as in figure 3D and electronic supplementary material, figure 1C), akin to that of adults [7,39,53,55,56,59]. Also, dinosaur tooth implantation (e.g. figure 3B) differs from the more symmetrical bone architecture around the teeth found in adult crocodylians and mammals (e.g. figure 3D) [7,17,39]. Conversely, toothed birds independently acquired a symmetric tooth implantation [60,61], so that toothed birds would be the only truly thecodont dinosaurs for Bertin *et al*. [12]. The term ‘subthecodonty’ (as illustrated by fig. 2B of [12] and fig. 1B of [17]) has been used to describe teeth with asymmetrical implantation, i.e. anisothecodont as proposed here (figure 3A,B), but also those with shallow sockets [4,57,58]. This latter use is, however, either very subjective or in need of a quantitative definition, and is not further discussed here.

In line with the new classification proposed here, examples of thecodonty are found across Amniota, including ichthyosaurs, mosasaurs, stem-mammals (e.g. non-mammalian synapsids), mammals, archosaurs and their close relatives. Most of these taxa have an ankylosed mode of tooth attachment, but also teeth implanted in sockets and, even for a short period, held in place by a network of periodontal ligament, as evidenced by the presence of Sharpey’s fibres in the cementum and alveolar bone (electronic supplementary material, figure 1B2) [5,7–9]. In fact, the time a tooth spends attached only by ligament strongly varies among taxa traditionally considered ‘ankylothecodonts’ [8,9,48].

## 5. Reinterpreting terms and the phylogenetic implications of the new nomenclature

As demonstrated, definitions for tooth attachment and implantation accumulate historical baggage that can lead to misinterpretations and the misuse of dental traits in phylogenies. Furthermore, due to conflations between tooth implantation and attachment for over a century, the periodontium of many reptiles has been associated with ‘bone of attachment’ (e.g. [62–67]) and even incorporated into diverse phylogenies as an anatomical character: e.g. *‘Tooth implantation: (0) free at the base of the tooth; (1) teeth fused to the bone of attachment at the base*’ [62,68–70]. However, ‘bone of attachment’ represents a historical misconception that should be avoided. Likewise, the term ‘ankylothecodonty’, frequently used to describe reptile periodontia (e.g. [12,62–70]), should be abandoned from the literature and instead simply be recognized as a type of thecodonty (as in figure 3)—a term safeguarded exclusively to describe teeth embedded within discrete sockets in the jaw, *sensu* Owen [25].

We also recognize that some taxa exhibit an intermediate condition (e.g. silesaurids, therocephalians, and many stem-mammals), in which both gomphosis and ankylosis are simultaneously present in different teeth of the same individual (e.g. figure 2C [8,9]). For this reason, when analysing tooth attachment characters, the shifts in timing and sequence of dental ontogeny are the traits that should be traced across phylogenies, thus identifying the mechanism leading to the transition from the ancestral rapid mineralization (ankylosis) to permanent gomphosis. The intermediate cases can be defined as thecodont taxa with a slower time of dental fusion, so that they simultaneously have ankylosed and non-ankylosed (gomphosis) teeth along the jaw for an extended period of their lives. Accordingly, using the character states defined by LeBlanc *et al*. [8]—(0), rapid ankylosis; (1), delayed ankylosis; and (2), permanent gomphosis—may be a useful approach to map and identify the evolution of different tooth attachment modes across amniote phylogeny.

For tooth implantation, traits related to the depth of the socket, the association (and position) of the odontogenetic organ/dental lamina with the overlying gum tissue (figure 3 and electronic supplementary material, figure 1C), and the related tooth replacement mode (underneath versus lingual to the functional tooth) are more suitable characters to use in phylogenetic analyses.

## 6. Concluding remarks

Gomphosis and ankylosis are two extremes of the same spectrum of tooth development. Heterochronic changes in the timing and extent of mineralization, not convergent evolution to mammal-like attachment tissues, led to the independent evolution of permanent gomphosis across independent clades within Amniota [8,9]. Regardless of the pace of mineralization, we recognize a shared developmental mechanism among teeth that form sockets, whether these sockets are asymmetrical or symmetrical. Accordingly, we propose that the term ‘thecodont’ should be used with reference to this shared amniote trait, and the terms ‘anisothecodont’ and ‘isothecodont’ with respect to variations in the symmetry of the sockets relative to the jawbone (figure 3). Across amniotes, synapsids (including humans), archosaurs (including hatchling crocodylians and dinosaurs) and their close relatives are all thecodonts. They are thecodonts not because of the status of their periodontal ligament mineralization or the symmetry (or depth) of their alveoli, but because they share that ancestral amniote condition that Owen [25] had already observed: their teeth are implanted in sockets within the jaw.

As demonstrated here, ‘bone of attachment’ does not accurately represent the nature of the tissues present in teeth ankylosed to the bone (figure 2 and electronic supplementary material, figure 1), despite being consistently mentioned in the contemporary literature (e.g. [12,62–69]). Therefore, we advocate for the permanent removal of the terms ‘bone of attachment’ and ‘ankylothecodont/ankylothecodonty’ from the scientific vocabulary. Indeed, thecodont teeth can be ankylosed to the bone (figure 3A,C), as an attachment mode opposite to gomphosis (figure 3B,D), as well as set in asymmetrical alveoli, i.e. anisothecodont (figure 3A,B), as opposed to isothecodont (figure 3C,D).

## Data Availability

This article has no additional data. The raw histological images used to generate the figures in the main article and electronic supplementary material are available from the Dryad Digital Repository [71]. Supplementary material is available online [72].
